# Macromolecular Crowding Induces Holo α-Lactalbumin Aggregation by Converting to Its Apo Form

**DOI:** 10.1371/journal.pone.0114029

**Published:** 2014-12-01

**Authors:** Shruti Mittal, Laishram Rajendrakumar Singh

**Affiliations:** Dr. B. R. Ambedkar Center for Biomedical Research, University of Delhi, Delhi, India; University of Hyderabad, India

## Abstract

Macromolecular crowding has been shown to have an exacerbating effect on the aggregation propensity of amyloidogenic proteins; while having an inhibitory effect on the non-amyloidogenic proteins. However, the results concerning aggregation propensity of non-amyloidogenic proteins have not been convincing due to the contrasting effect on holo-LA, which despite being a non-amyloidogenic protein was observed to aggregate under crowded conditions. In the present study, we have extensively characterized the crowding-induced holo-LA aggregates and investigated the possible mechanism responsible for the aggregation process. We discovered that macromolecular crowding reduces the calcium binding affinity of holo-LA resulting in the formation of apo-LA (the calcium-depleted form of holo-LA) leading to aggregate formation. Another finding is that calcium acts as a chaperone capable of inhibiting and dissociating crowding-induced holo-LA aggregates. The study has a direct implication to Alzheimer Disease as the results invoke a new mechanism to prevent A*β* fibrillation.

## Introduction

The primary sequence of a globular protein is important in making the unfolded proteins *en-route* to its native, functional three-dimensional structure *via* intramolecular interactions [1], [2]. However, proteins (if not properly folded) often have a tendency to interact intermolecularly in the course of protein unfolding, refolding, or de novo folding, leading to the formation of disordered aggregates to amyloids. Presently, protein aggregation is of intense medical interest since the deposition of protein aggregates *in vivo* is related to the pathogenesis of many human diseases including neurodegenerative, metabolic, cardiovascular disorders etc [3], [4], [5], [6], [7], [8], [9], [10], [11]. It has been shown that proteins involved in such diseases have different sequences and tertiary structures [12], [13], [14]. In addition, most proteins or peptides not associated with such diseases can also form aggregates under specific conditions, suggesting that the ability to form aggregates is a common property of all proteins or polypeptide chains [15]. Considerable effort has so far been made to achieve the fundamental understanding of the basic cause and factors affecting the aggregation processes that lead to disease development [13], [14]. However, most solvent environments used traditionally to study protein aggregation processes were highly dilute (e.g., Tris-HCl or phosphate buffer) as compared to the highly crowded, intracellular environment where proteins perform their biological functions [16], [17]. Indeed, the cells interior is known to be densely populated due to the presence of soluble and insoluble macromolecules (proteins, nucleic acids, ribosomes and carbohydrates etc) [16], [18], which together make the intracellular environment “crowded” or “volume-occupied” rather than “concentrated” [18], [19], [20]. These macromolecules collectively occupy ∼10–40% (a substantial fraction of the intracellular space) of the total fluid volume, thereby restricting the volume available to other macromolecules present [18]. Crowded environment therefore, results in altered biological processes including protein folding and aggregation as compared to those under dilute buffers [18]. Until recently, this difference was mainly accounted by the excluded volume effect [21], [22]. However, a great deal of recent work [23], [24] shows that in addition to excluded volume effect, soft interactions also play an important role in determining macromolecular properties.

Effect of macromolecular crowding on protein aggregation has been extensively studied using a variety of different macromolecules as potential macromolecular crowding agents: proteins, polysaccharides and synthetic polymers [25], [26], [27]. The current understanding is that macromolecular crowding increases the rate and extent of protein aggregation and fibril formation [25], [28], [29], [30], [31]. Recent developments further evidenced that macromolecular crowding has opposite consequences on the aggregation propensities of amyloidogenic and non-amyloidogenic proteins [32]. It has been suggested that macromolecular crowding has an enhancing effect on the aggregation propensity of amyloidogenic proteins, while having an inhibitory effect on the non-amyloidogenic proteins. However, in a recent work our laboratory [33] observed that macromolecular crowding induces aggregation (or precipitation) of holo α-lactalbumin (holo-LA), a non-amyloidogenic protein under slightly acidic conditions (pH 4.0–5.0). The contrasting effect of crowding on holo-LA compared to other non-amyloidogenic proteins prompted us to uncover the insights of holo-LA aggregation under crowded conditions. In this spirit, we have characterized the crowding-induced holo-LA aggregates and investigated the possible mechanism responsible for the aggregation process. We discovered that macromolecular crowding induces holo-LA aggregation due to reduction in its calcium-binding affinity. We also found that calcium acts as a chaperone capable of inhibiting and dissociating crowding-induced holo-LA aggregates. Based on our results, we further propose that increasing the expression of calcium-binding proteins might help to reduce Alzheimer Disease (AD) pathogenesis by offsetting the aggregation of Aβ.

## Materials and Methods

### 1. Materials

Commercially lyophilized preparation of holo α-Lactalbumin (from bovine milk) was purchased from Sigma Chemical Co (St Louis, MO, USA). Ficoll 70, trichloroacetic acid (TCA), Thioflavin T (ThT), 8-Anilino-1-naphthalenesulfonic acid ammonium salt (ANS), Ethylenediaminetetraacetic acid (EDTA) and sodium salt of cacodylic acid were also obtained from Sigma Chemical Co. Potassium chloride (KCl) and sodium acetate were obtained from Merck. Calcium chloride was obtained from G-Biosciences. These and other chemicals, which were of analytical grade, were used without further purification.

### 2. Analytical procedures

Holo-LA solution was dialyzed extensively against 0.1 M KCl at pH 7.0 in cold (∼4°C). Apo-α-lactalbumin (apo-LA) was prepared by adding 3.5 mM EDTA (pH 7.0) to the solution of holo-protein. Protein stock solutions were filtered using 0.22-µm millipore filter paper. Both the proteins gave a single band during polyacrylamide gel electrophoresis. Concentration of the protein solutions were determined experimentally using *ε*, the molar absorption coefficient value of 29,210 M^−1^ cm^−1^ at 280 nm [34]. All solutions for optical measurements were prepared in 0.05 M sodium acetate buffer (pH 4.5). Special care was taken to thoroughly mix all solutions due to the high viscosity of Ficoll 70.

### 3. Light scattering measurements

#### 3.1 Temperature-dependent aggregation

Temperature-dependent holo-LA and apo-LA aggregation were followed by monitoring the light scattering intensity i.e., the apparent absorbance at 400 nm of the sample solutions (pH 4.5) in a Jasco V-660 UV/Visible spectrophotometer equipped with a Peltier-type temperature controller at a heating rate of 1°C per minute. This scan rate was found to provide adequate time for equilibration. Each sample was heated from 20°C to 80°C. About 600 data points of each transition curve were collected. Necessary blanks were subtracted. Measurements were repeated at least three times.

#### 3.2 Analysis of temperature-dependent aggregation parameters

The sigmoidal dependence of the light scattering intensity on temperature was analyzed using the following equation, 
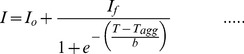
(1)where *I* is the light scattering intensity at temperature *T*, and *T*_agg_ is the temperature at 50% maximal light scattering, *I*_o_ and *I*_f_ represent the initial base line and final plateau line, respectively. *b* is a constant. Thus, the temperature at which aggregation (increase in light scattering intensity) begins (*T*_i_) is given by *T*_agg_-2*b* [35].

#### 3.3 Time-dependent aggregation

50 µM holo-LA in the presence of 400 g/l Ficoll 70 (46.4°C; pH 4.5) was placed into 10 mm path length quartz cuvette and the aggregation process was followed by monitoring the change in light scattering at 400 nm using a V-660 UV/Vis Spectrophotometer. After 4 hours, equal volume of either acetate buffer or calcium chloride was added to the aggregated sample and to their respective blanks. Calcium chloride was added such that the final concentration in the sample was 100 mM. Necessary blanks were subtracted. Measurements were repeated three times.

### 4. Fluorescence Measurements

#### 4.1 ANS binding assay

50 µM holo-LA under crowded conditions (in the absence and presence of varying [CaCl_2_], the molar concentration of calcium chloride) was kept at different temperatures. Aliquots of each of the holo-LA samples incubated in this manner was diluted and brought to room temperature in acetate buffer containing ANS, such that the final protein and ANS concentration were 0.5 µM and 20 µM, respectively. Concentration of ANS was determined experimentally using *ε*, the molar absorption coefficient value of 5,000 M^−1^ cm^−1^ at 350 nm [36] and was filtered before use to remove insoluble particles. Samples were excited at 360 nm and ANS binding spectra were recorded in a 5 mm quartz cell between 400 and 600 nm at 25°C with an excitation and an emission slit width of 10 nm at a scanning speed of 100 nm/min in a Perkin-Elmer LS-55 Spectrofluorimeter. Necessary blanks were subtracted.

#### 4.2 ThT binding assay

50 µM holo-LA under crowded conditions was kept at different temperatures. Aliquots of each of the holo-LA samples incubated in this manner was diluted and brought to room temperature in acetate buffer containing ThT, such that the final protein and ThT concentration were 5 µM and 25 µM, respectively. Concentration of ThT was determined experimentally using *ε*, the molar absorption coefficient value of 26,620 M^−1^ cm^−1^ at 416 nm [37] and was filtered before use to remove insoluble particles. ThT binding was measured in a Perkin-Elmer LS-55 Spectrofluorimeter in a 5 mm quartz cell with both excitation and emission slit width set at 10 nm. For ThT binding measurements, samples were excited at 450 nm and emission spectra were recorded in the wavelength region 470-570 nm at a scanning speed of 100 nm/min. Necessary blanks were subtracted.

### 5. Thermal denaturation studies

Thermal denaturation studies were carried out in a Jasco V-660 UV/Visible spectrophotometer equipped with a Peltier-type temperature controller at a heating rate of 1°C per minute. This scan rate was found to provide adequate time for equilibration. Each sample was heated from 20°C to 85°C. The change in absorbance with increasing temperature was followed at 295 nm (pH 4.5) using appropriate blanks. About 650 data points of each transition curve were collected. Measurements were repeated at least three times. After denaturation, the protein sample was immediately cooled down to measure reversibility of the reaction. Each heat-induced transition curve was analyzed for *T*_m_ and *ΔH*_m_ using a non-linear least-squares method according to the relation,
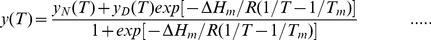
(2)where *y*(*T*) is the optical property at temperature *T* (Kelvin), *y*_N_(*T*) and *y*_D_(*T*) are the optical properties of the native and denatured protein molecules at *T* K, respectively, and *R* is the gas constant. In the analysis of the transition curve, it was assumed that a parabolic function describes the dependence of the optical properties of the native and denatured protein molecules (i.e. *y*_N_(*T*)  =  a_N_+b_N_ *T*+c_N_ *T* ^2^ and *y*_D_(*T*)  =  a_D_+b_D_ *T*+c_D_ *T* ^2^, where a_N_, b_N_, c_N_, a_D_, b_D_, and c_D_ are temperature-independent coefficients) [38].

### 6. Protein quantification

After heat-induced denaturation of holo-LA samples in the presence of varying concentrations of calcium chloride under crowded conditions (400 g/l Ficoll 70), samples were centrifuged for 30 minutes at 15,600×g in an Eppendorf 5415 R Centrifuge (Eppendorf AG, Hamburg, Germany). The pellets were washed three times with 0.05 M acetate buffer, pH 4.5, and the pellets were stored at −20°C for imaging purposes. The soluble protein in the supernatants was precipitated with 5% TCA, and the pellets obtained after centrifugation (13,000 rpm for 30 minutes) were washed three times to remove any remaining crowder or calcium chloride. Protein concentration was measured at 280 nm spectrophotometrically.

### 7. Transmission electron microscopy

Pellets obtained above were re-dissolved in double distilled water. A 10 µl sample was placed on a formvar-coated copper grid and left at room temperature for 5 minutes. The grids were negatively stained with 1% uranyl acetate solution for another 2 minutes before examination using a Tecnai G2-200 kV HRTA transmission electron microscopy (FEI, Netherland) operating at a voltage of 200 kV.

### 8. Circular Dichroism measurements

CD measurements were made in a Jasco J-810 Spectropolarimeter equipped with a Peltier-type temperature controller with three accumulations at 45°C. Protein concentration used for the CD measurements was 50 µM. Spectra of holo-LA were recorded under dilute conditions; in the presence of 1.2 mM EDTA; 300 g/l Ficoll 70 and 300 g/l Ficoll 70 with 100 mM CaCl_2_. Cells of 0.1 and 1.0 cm path length were used for the measurements of the far- and near-UV spectra, respectively. Necessary blanks were subtracted. The CD instrument was routinely calibrated with D-10-camphorsulfonic acid.

## Results

**Figure 1:**
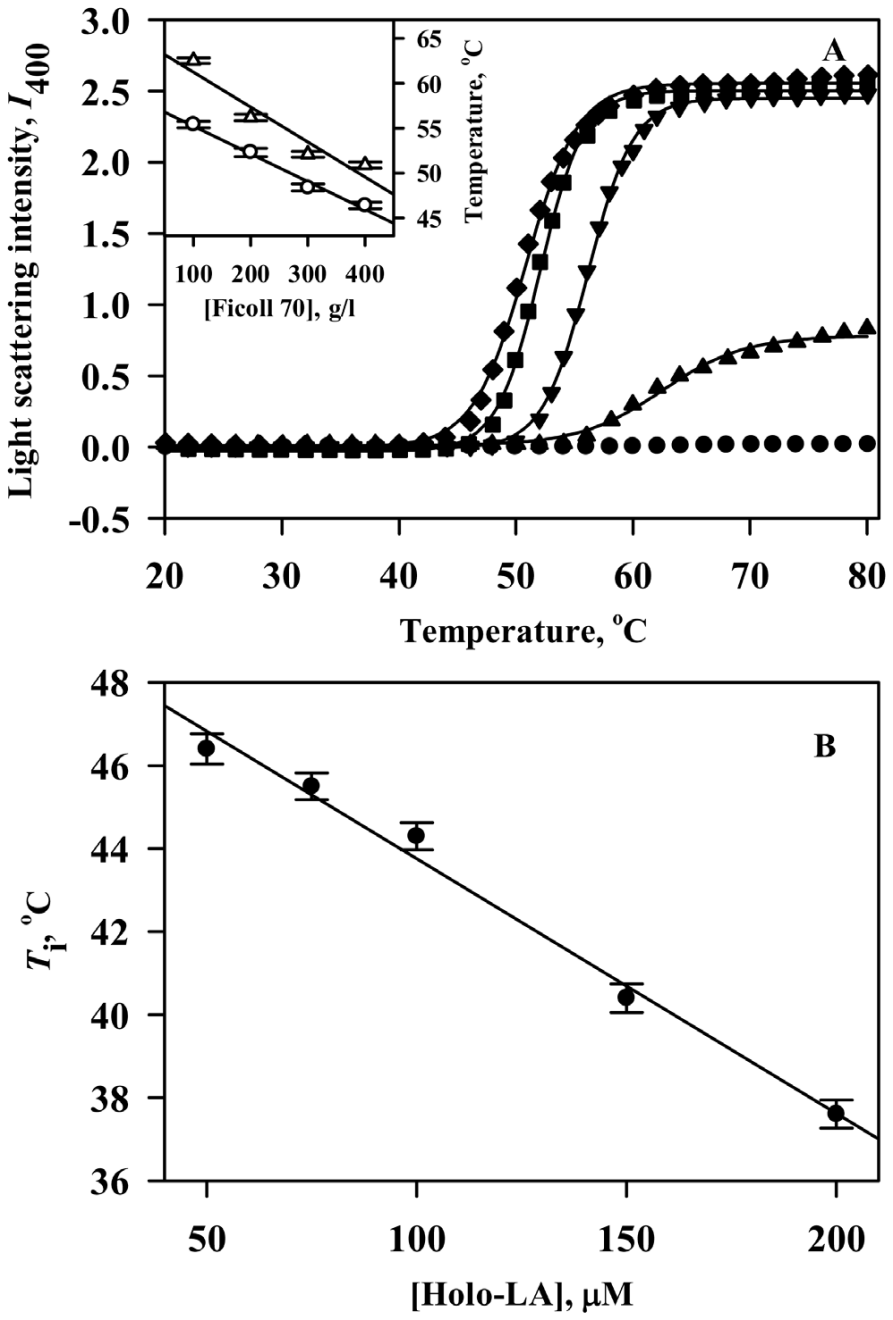
Temperature-dependent aggregation profiles of holo-LA. (A) The temperature dependence of the light scattering intensity at 400 nm of holo-LA in the absence and presence of varying [Ficoll 70], the molar concentration of Ficoll 70. The lines represent the best-fits (using Equation 1) of data obtained in the presence of 0 g/l (solid circle), 100 g/l (solid triangle), 200 g/l (solid inverted triangle), 300 g/l (solid square) and 400 g/l (solid diamond) Ficoll 70. *Inset* shows the plot of *T*_agg_ (open triangle) and *T*_i_ (open circle) *versus* [Ficoll 70]. (B) Plot of *T*_i_ versus [holo-LA].

Macromolecular crowding has been reported to have opposite consequences on the aggregation propensity of amyloidogenic (enhancing effect) and non-amyloidogenic proteins (inhibitory effect) [32]. However, in our recent study [33], holo-LA (a non-amyloidogenic protein) was observed to aggregate under crowded conditions. To unfold the factors responsible for holo-LA aggregation under crowded conditions, we have first of all investigated the effect of various concentrations of Ficoll 70 on the temperature-dependent aggregation of holo-LA using light scattering as the probe. Figure 1A shows holo-LA aggregation profiles at pH 4.5 monitored by observing change in light scattering intensity at 400 nm (*I*_400_) as a function of temperature in the absence and presence of varying amounts of Ficoll 70. It is seen in this figure that under dilute conditions, no increase in the light scattering intensity was observed in the temperature regime (20°C–80°C) while there was a dramatic increase in the light scattering intensity under crowded conditions. Control experiments were performed to ensure that Ficoll 70 had no influence on the light scattering intensity. The temperature-dependent aggregation profiles (dependence of *I* on temperature) obtained under crowded conditions were analyzed using Equation (1) to yield the three parameters- *I*_f_ (light scattering at the final plateau line), *T*_agg_ (temperature at 50% maximal light scattering) and *T*_i_ (temperature at which increase in light scattering intensity begins) (summarized in Table 1). It is seen in this table that *I*_f_ increases with increasing crowder concentration indicating an increase in the extent of aggregation. In addition, *T*_agg_ and *T*_i_ are observed to decrease in a crowder concentration dependent manner suggesting that the native state of the protein has been destabilized by Ficoll 70 subsequently resulting in aggregation initiation from lower temperatures. For instance, aggregation initiates at 46.4°C in the presence of 400 g/l Ficoll 70 instead of 55.4°C at 100 g/l Ficoll 70 (Figure 1A: *inset*). For most protein aggregation systems, increasing protein concentration results in increased aggregation. Therefore, aggregation profiles of holo-LA (at different concentrations) in the presence of highest concentration of Ficoll 70 (400 g/l) were also measured by monitoring change in light scattering intensity at 400 nm as a function of temperature (Figure S1). The curves were analyzed using Equation (1) to yield *T*_i_ which was observed to decrease linearly as a function of [holo-LA], the molar concentration of holo-LA (Figure 1B). Results indicate that Ficoll 70 decreases the native state stability of holo-LA. It is important to note that *T*_i_ drops down to 38°C (near physiological temperature) in the presence of 200 µM holo-LA suggesting that aggregation can be made to initiate at lower temperatures also.

**Table 1:** Parameters of temperature-dependent holo-LA aggregation in the presence of varying Ficoll 70 concentrations^a^.

[Ficoll 70] (g/l)	*I*_f_	*T*_agg_ (°C)	*T*_i_ (°C)
100	0.77	62.5	55.4
200	2.46	56.2	52.3
300	2.53	52.1	48.4
400	2.55	50.9	46.4

a Errors in *I*_f_, *T*_agg_ and *T*_i_ are 6–8%, 0.5–0.7% and 0.6–0.9%, respectively.

**Figure 2:**
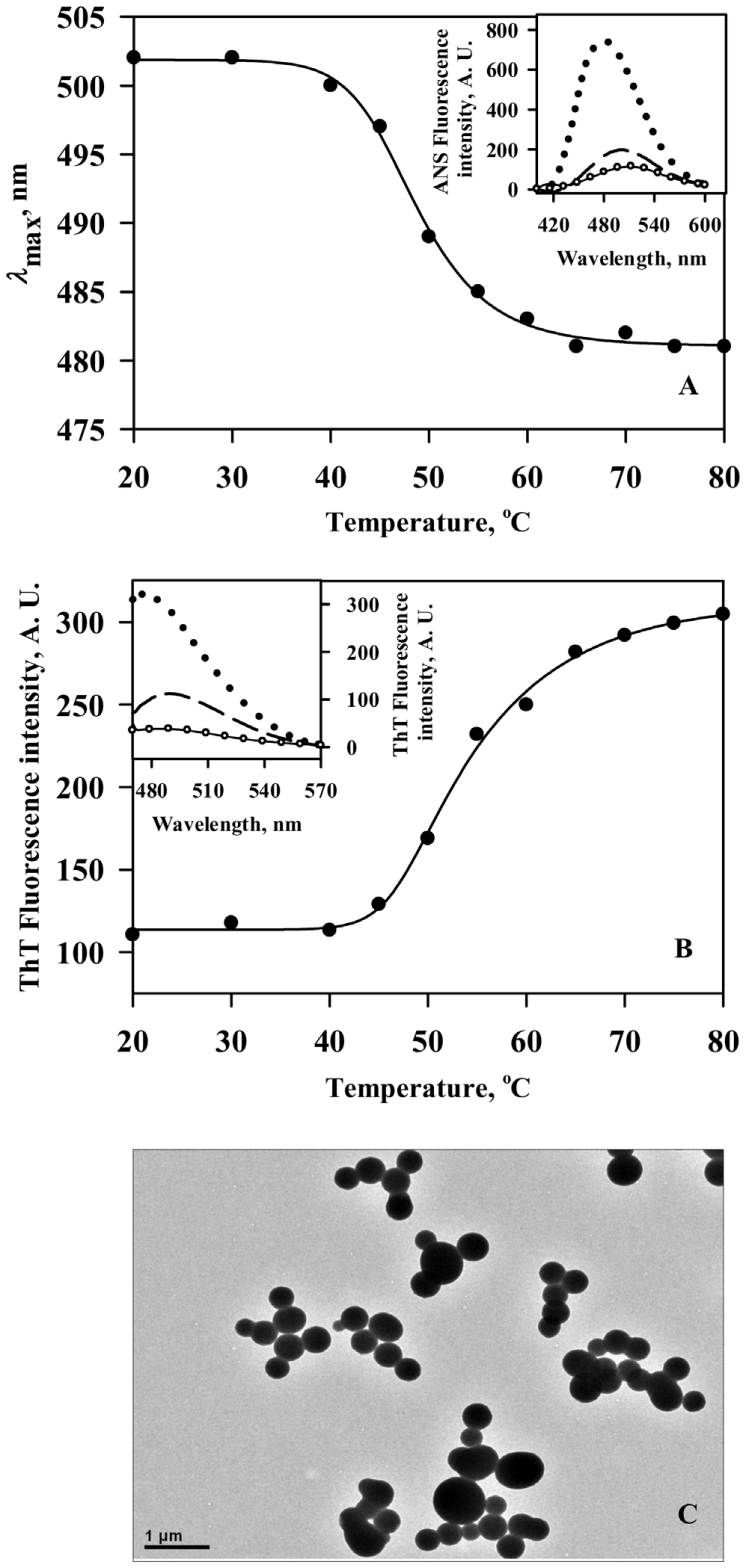
Characterization of the Ficoll 70-induced holo-LA aggregates. (A) Plot of ANS binding profile (*λ*_max_ *versus* temperature) of holo-LA aggregates formed in the presence of Ficoll 70 at different temperatures. *Inset* shows the representative ANS binding profiles of holo-LA in the absence (20°C: solid line; 80°C: open circle) and presence of Ficoll 70 (20°C: dashed line; 80°C: dotted line). (B) Plot of ThT binding profile (ThT fluorescence intensity at 485 nm *versus* temperature) of holo-LA aggregates formed in the presence of Ficoll 70 at different temperatures. *Inset* shows the representative ThT binding spectra of holo-LA aggregates formed in the absence (20°C: solid line; 80°C: open circle) and presence of Ficoll 70 (20°C: dashed line; 80°C: dotted line). (C) Electron micrograph of holo-LA aggregates following heat-induced denaturation in the presence of 400 g/l Ficoll 70.

Figure 2A illustrates ANS binding profile of the holo-LA aggregates formed in the presence of 400 g/l Ficoll 70 (pH 4.5) at different temperatures. Representative spectra (at extreme temperatures) are also shown in the inset of Figure 2A. Control spectra at 20°C and 80°C are also shown. It is seen in this figure that there is no significant change in ANS fluorescence intensity at different temperatures under dilute conditions. However, for holo-LA aggregates formed under crowded conditions, the ANS binding spectra are observed to be blue-shifted (∼20 nm) accompanied by a remarkable increase in fluorescence intensity with increasing temperature from 20°C to 80°C (Figure 2A: *inset*). ThT fluorescence intensity at 485 nm (which reports the presence of amyloids) was found to remain unaffected at higher temperature under dilute conditions in contrast to the increase in ThT fluorescence observed for holo-LA aggregates formed under crowded conditions (Figure 2B and *inset* of Figure 2B). Electron micrographs of holo-LA aggregates formed in the presence of 400 g/l Ficoll 70 (Figure 2C) revealed the presence of spherical aggregates with no evidence of amyloid fibrils.

**Figure 3:**
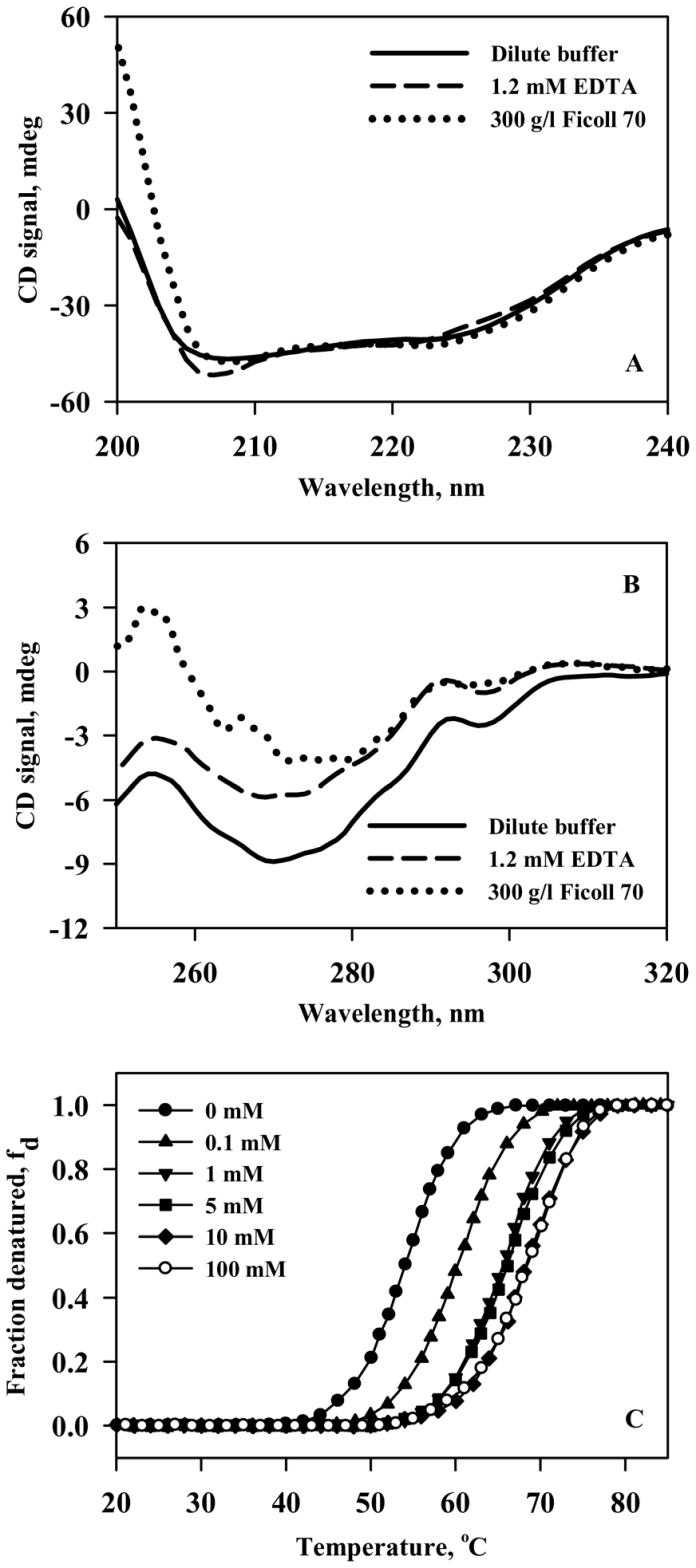
Reduction in the calcium-binding affinity of holo-LA in the presence of Ficoll 70. (A) Far-UV CD spectra of holo-LA at 45°C under different solvent conditions: dilute (solid line); 1.2 mM EDTA (dashed line) and 300 g/l Ficoll 70 (dotted line). (B) Near-UV CD spectra of holo-LA at 45°C under different solvent conditions: dilute (solid line); 1.2 mM EDTA (dashed line) and 300 g/l Ficoll 70 (dotted line). (C) Representative thermal denaturation profiles of holo-LA in the presence of varying calcium chloride concentrations under dilute (solid symbols) and crowded (open symbol) conditions. In order to maintain clarity, some transition curves have not been shown.

To uncover the conformational changes brought about in holo-LA (responsible for aggregation) by Ficoll 70, secondary and tertiary structures were measured at 45°C (pH 4.5) in the absence and presence of 300 g/l Ficoll 70 instead at 48°C, the temperature at which aggregation initiates in the presence of 300 g/l Ficoll 70. The temperature used for characterizing conformational changes in holo-LA under crowded conditions was chosen such that the protein sample is completely devoid of aggregates (as evidenced in Figure 1A) during the time period of conformational measurement. In terms of secondary structure (Figure 3A), holo-LA is observed to be insignificantly affected due to the presence of Ficoll 70 relative to that under dilute conditions. However, tertiary structure measurements (Figure 3B) revealed that holo-LA experiences loss in tertiary interactions under crowded conditions (as manifested in the reduced CD signal relative to that under dilute conditions). Results suggest that the conformation of intact holo-protein in the presence of Ficoll 70 becomes similar to that of the apo-protein in dilute buffer at 45°C. Figure S2 shows profiles of apo-LA aggregation monitored by observing change in light scattering intensity at 400 nm in the absence and presence of 400 g/l Ficoll 70 (pH 4.5) as a function of temperature. It is seen in the figure that the light scattering intensity remains unaffected under dilute conditions in contrast to the marked increase in the presence of Ficoll 70. Above evidences indicate that holo-LA undergoes aggregation under crowded conditions most probably due to conversion of holo-LA to apo-form. We have further measured the heat-induced denaturation of holo-LA in the presence of different calcium chloride concentrations (0–400 mM) in the absence and presence of 400 g/l Ficoll 70 at pH 4.5 by following change in absorbance at 295 nm as a function of temperature (Figure 3C). Denaturation of holo-LA was reversible in the entire range of [CaCl_2_] under dilute as well as crowded conditions. It is important to note that we observed visible precipitation of holo-LA in the presence of calcium chloride concentrations lower than 100 mM under crowded conditions. Therefore, we could not obtain reversible heat-induced transition curves under these experimental conditions. Each denaturation curve of holo-LA at a given [CaCl_2_] under dilute and crowded conditions was analyzed for *T*_m_ (midpoint of denaturation) and Δ*H*_m_ (denaturational enthalpy change at *T*_m_) using a nonlinear least-squares method that involves fitting the entire data of the transition curve to Equation (2) with all eight free parameters (a_N_, b_N_, c_N_, a_D_, b_D_, c_D_, Δ*H*_m_ and *T*_m_). Table 2 shows values of *T*_m_ and Δ*H*_m_ of holo-LA in the absence and presence of different [CaCl_2_] under dilute and crowded conditions. It is seen in this table that holo-LA in dilute buffer requires only 10 mM calcium chloride to achieve a *T*_m_ of 68°C (*T*_m_ of the holo-protein in the absence of Ficoll 70 at physiological pH) as compared to 100 mM calcium chloride under crowded conditions to achieve the same stability. The result clearly indicates that the calcium-binding affinity to holo-LA under dilute and crowded conditions is different.

**Table 2:** Thermodynamic parameters of holo-LA in the presence of varying calcium chloride concentrations under dilute and crowded conditions^a^.

[CaCl_2_]	Dilute		Crowded (400 g/l Ficoll 70)	
(mM)	*T*_m_ (°C)	*ΔH*_m_ (kcal mol^−1^)	*T*_m_ (°C)	*ΔH*_m_ (kcal mol^−1^)
0.0	54.4	53	ND	ND
0.1	60.2	64	ND	ND
1.0	65.3	72	ND	ND
5.0	66.6	74	ND	ND
10.0	68.2	77	ND	ND
25.0	68.3	79	ND	ND
50.0	68.8	80	ND	ND
100.0	69.0	78	68.8	76
200.0	68.2	78	68.3	80
400.0	68.5	77	68.6	79

a Errors in *T*_m_ and Δ*H*_m_ are 0.3–0.8% and 5–8%, respectively.

ND Not determined.

**Figure 4:**
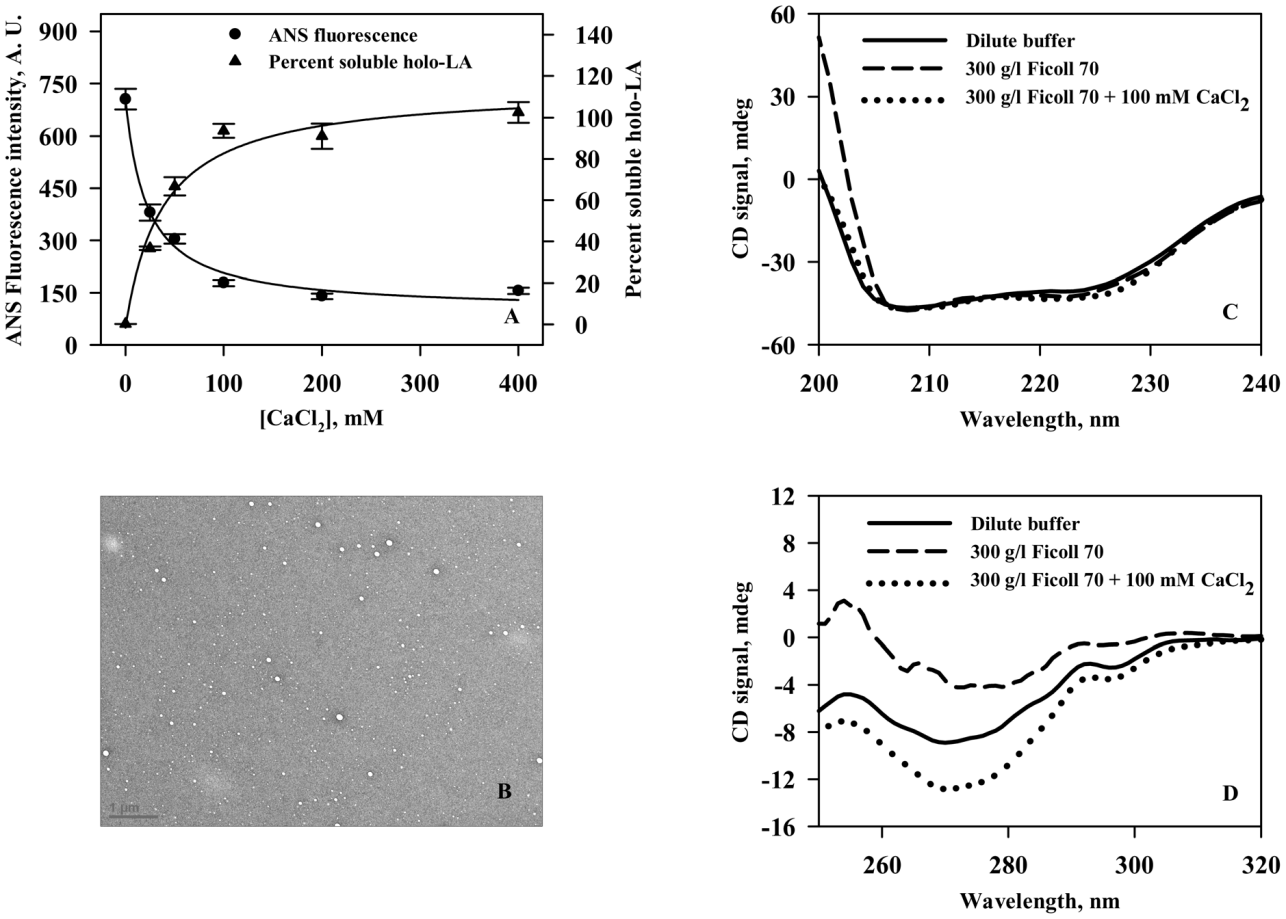
Calcium inhibits crowding-induced holo-LA aggregation. (A) Plot of ANS fluorescence intensity (at 473 nm) *versus* [CaCl_2_] (solid circle) and percent soluble protein *versus* [CaCl_2_] (solid triangle) under Ficoll 70 treated conditions. (B) Electron micrograph of holo-LA following heat-induced denaturation in the presence of 400 g/l Ficoll 70 and 100 mM calcium chloride. (C) Far-UV CD spectra of holo-LA at 45°C under different solvent conditions: dilute (solid line); 300 g/l Ficoll 70 (dashed line) and 300 g/l Ficoll 70 plus 100 mM CaCl_2_ (dotted line). (D) Near-UV CD spectra of holo-LA at 45°C under solvent conditions: dilute (solid line); 300 g/l Ficoll 70 (dashed line) and 300 g/l Ficoll 70 plus 100 mM CaCl_2_ (dotted line).

We have further investigated the ability of calcium to inhibit the crowding-induced aggregate formation using a variety of probes, namely, ANS binding assay, centrifugation (to quantitate soluble protein), transmission electron microscopy and circular dichroism (Figure 4). It is seen in Figure 4 that holo-LA aggregation decreases in a calcium chloride concentration dependent manner and the percent soluble protein concomitantly increases with increasing concentrations of calcium chloride (Figure 4A). Electron micrograph represented in Figure 4B further confirmed calcium chloride-mediated inhibition of Ficoll 70-induced holo-LA aggregation. Figures 4C and 4D show that there is a gain in tertiary interactions of holo-LA in the presence of calcium chloride under crowded conditions while leaving the secondary structure insignificantly perturbed.

**Figure 5:**
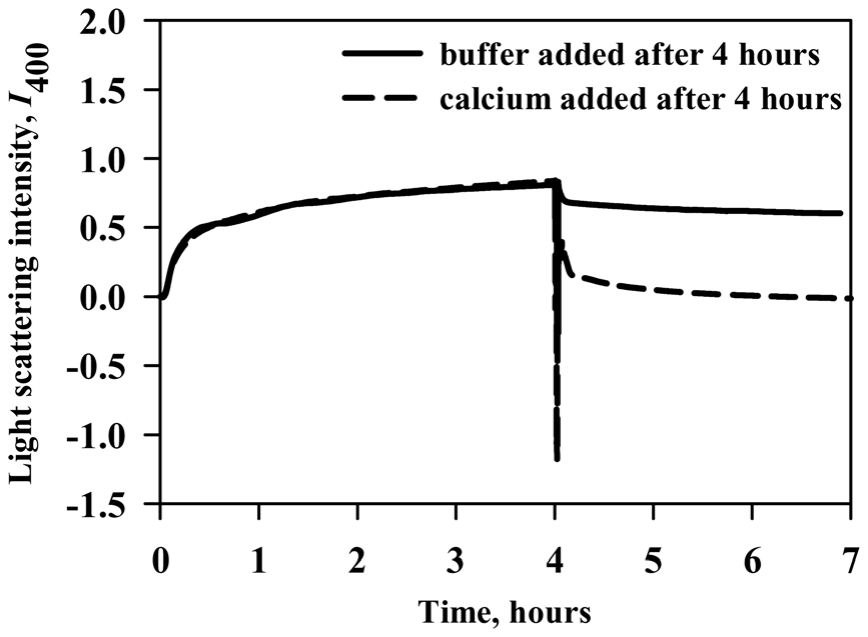
Calcium reverses crowding-induced holo-LA aggregates. Aggregation kinetics of holo-LA monitored by observing change in light scattering intensity at 400 nm in the presence of 400 g/l Ficoll 70. After 4 hours, calcium chloride was added to the protein-crowder system.

We then examined if calcium chloride could also reverse holo-LA aggregation using light scattering (at 400 nm) as a tool (Figure 5). For this, holo-LA aggregation in the presence of 400 g/l Ficoll 70 was allowed to saturate by incubation at 46.4°C (pH 4.5) for 4 hours. We use this temperature as it has been observed in Figure 1 and Table 1 that aggregation initiates at 46.4°C in the presence of 400 g/l Ficoll 70. After saturation, equal volumes of either acetate buffer or calcium chloride were added to the aggregated holo-LA sample. It is seen in the figure that upon addition of buffer, there was a slight decrease in the light scattering intensity which is attributed to the dilution of holo-LA aggregates. However, when excess calcium chloride (working concentration of 100 mM) was added, there was a rapid decrease in light scattering intensity ultimately touching the zero baseline (with no visible precipitates) suggesting the absence of aggregates. Control experiments were also performed to ensure that Ficoll 70 had no influence on light scattering intensity (Figure S3). Our results indicate that calcium could not only inhibit but could also reverse crowding-induced holo-LA aggregation.

## Discussion

In our previous study, macromolecular crowding (using Ficoll 70) was observed to induce aggregation (and precipitation) of holo-LA (a non-amyloidogenic protein) instead of expected increase in the thermodynamic stability at lower pH values (4.0–5.0) [33]. Keeping in mind that the pH range is physiological for many cell organelles and organisms [39], [40], it is important to investigate the aggregation behaviour of holo-LA under crowded conditions at such low pH values. Using light scattering as a tool, we have monitored the temperature dependence of holo-LA aggregation in the presence of varying concentrations of Ficoll 70 at pH 4.5 (Figure 1A). The analysis of the temperature-dependent aggregation profiles and the parameters thus obtained (summarized in Table 1) revealed that (i) the extent of aggregation (*I*_f_) increases with increasing crowder-concentration, and (ii) mid-point of heat-induced aggregation (*T*_agg_) and aggregation initiation temperature (*T*_i_) were shifted to lower temperatures in a crowder-concentration dependent manner indicating that aggregation of holo-LA is due to destabilization of the native state of holo-LA by Ficoll 70. Since, protein aggregation results from accumulation of aggregation-prone intermediates [41], the destabilization of the native state in the presence of Ficoll 70 is expected to result in the increased production of aggregation-prone intermediates. If this is true, then increase in the protein concentration should result in increased accumulation of aggregation-prone intermediates consequently leading to a decrease in *T*_i_. To investigate for this, temperature-dependent aggregation profiles of holo-LA (at increasing concentrations) in the presence of 400 g/L Ficoll 70 were measured. The estimated *T*_i_ values (using Equation 1) are plotted against varying holo-LA concentrations in Figure 1B. The decrease in *T*_i_ as a function of [holo-LA] is a clear indication of the destabilization of the native state stability by Ficoll 70. Characterization of the nature of aggregates, using ANS and ThT binding assays and TEM images revealed the formation of spherical aggregates, but not amyloids (Figure 2). Binding of ThT, but absence of amyloid fibrils (as evidenced by electron micrograph) might be due to the exposure of beta-sheet structure present in native holo-LA. Taken together, we conclude that Ficoll 70 induces aggregation of holo-LA by destabilizing the native state resulting in the accumulation of aggregation-prone intermediates.

What factor is responsible for the native state destabilization by Ficoll 70? Holo-LA is a calcium-binding protein where calcium is known to play an important role in determining its structure and stability [42]. In addition, apo-LA (the calcium-depleted form of holo-LA) is known to have low thermodynamic stability relative to holo-LA [43], [44]. Therefore, it appears that reduction in the native state stability of holo-LA under crowded conditions might be a consequence of reduced calcium-binding affinity. To look into this possibility, we measured the conformational changes brought about by Ficoll 70 at 45°C (Figure 3). As evident in Figure 3, secondary and tertiary structures of holo-LA under crowded conditions behaves like the apo-form and retains most of its rigidity (due to no effect on the far-UV CD spectra in the presence of 300 mg/ml relative to that under dilute conditions) but becomes quite flexible (due to decrease in the near-UV CD signal in the presence of Ficoll 70) relative to that under dilute conditions. The results, therefore suggest that holo-LA undergoes a conformational transition to apo-form under crowded conditions. If this is indeed true, (i) apo-LA (like holo-LA) should also aggregate in the presence of Ficoll 70; and (ii) treatment with excess CaCl_2_ should inhibit Ficoll 70-induced holo-LA aggregation. To verify for the first speculation, temperature-dependent aggregation of apo-LA using light scattering (at 400 nm) was investigated in the absence and presence of 400 g/l Ficoll 70 (Figure S2). It is seen in the figure that the light scattering intensity remains unaffected under dilute conditions in contrast to the dramatic increase observed in the presence of Ficoll 70, thereby confirming that apo-LA aggregates under crowded condition. To investigate for the second speculation, we have measured the heat-induced denaturation of holo-LA in the presence of 400 g/l Ficoll 70 with varying [CaCl_2_] by monitoring change in absorbance at 295 nm (Figure 3C). Thermal denaturation profiles obtained were analyzed for *T*_m_ and Δ*H*_m_ (summarized in Table 2) using Equation (2). At least three important observations were made (i) the thermal denaturation profiles of holo-LA under crowded conditions were reversible in the presence of calcium chloride concentration of 100 mM and above; (ii) calcium chloride confers a thermodynamic stabilizing influence on holo-LA under both dilute and crowded conditions; and (iii) the calcium-binding affinity to holo-LA under crowded conditions is 10 times lower than that observed under dilute conditions. It has been known that macromolecular crowding increases the effective concentration of any small solute by decreasing its available volume through steric repulsion as a consequence of excluded volume effect [45], [46], [47]. We have, therefore calculated the effective concentration of calcium in the presence of 400 g/l Ficoll 70 by using appropriate equations reported earlier [19], [48]. We found that 400 mM CaCl_2_ under dilute conditions is equivalent to 540 mM CaCl_2_ in the presence of 400 g/l Ficoll 70. The results indicate that the calcium concentration required to maintain lactalbumin in holo-form under crowded intracellular environment has been underestimated through dilute *in vitro* investigations. Taken together, we conclude that macromolecular crowding decreases the calcium-binding affinity of holo-LA, and addition of calcium converts the irreversible heat-induced denaturation transitions to reversible ones.

In holo-LA, calcium co-ordination at the calcium binding loop involves the carboxylic oxygens of Asp82, Asp87 and Asp88; the carbonyl oxygens of Asp84 and Lys79 and two water molecules [49]. Particularly, the orientation of Asp87 relative to calcium ion is important in determining whether lactalbumin will be in a calcium-free or bound state [50]. It has also been reported earlier that the radius of holo-LA (and, hence structure exposition to the solvent) is more at low pH than at physiological pH [51]. Since, macromolecular crowding is known to stabilize proteins as a consequence of excluded volume effect by shifting the more expanded conformations to a folded, compact conformation [28], [52], the increase in the radius of the native state (due to low pH) will favor an increase in the volume excluded to the surrounding molecules. Therefore, it is possible that macromolecular crowding (Ficoll 70) distorts the orientation of Asp87 relative to calcium ion in an attempt to minimize the exposition of native state structure to the solvent resulting in reduced calcium-binding affinity. In addition to the role of volume exclusion, a great deal of recent work [53], [54] suggests that soft interactions between background and test molecules also play an important role in determining macromolecular properties under physiologically relevant conditions. In this case, soft interactions seem unlikely to have a significant contribution towards the observed effect. Because holo-LA has a pI ∼4.5–5.0 and thus will be negligibly charged at pH 4.5, thereby reducing the possibility of soft interactions with the background crowder molecules. However, we do not completely rule out the possible role of soft interactions in the observed effect.

We have further investigated if calcium chloride inhibits holo-LA aggregation in the presence of crowding agent, by ANS fluorescence and quantitation of soluble protein. As seen in Figure 4A, there is a decrease in the ANS fluorescence intensity with a concomitant increase in the percent soluble holo-LA as a function of [CaCl_2_] suggesting that calcium chloride prevents holo-LA aggregation under crowded conditions. Electron micrograph (Figure 4B) further confirmed the absence of holo-LA aggregates under crowded conditions in the presence of calcium chloride. To unveil the reason behind the calcium-mediated inhibition of crowding-induced aggregation, conformational measurements in the presence of calcium chloride under crowded conditions (Figures 4C and 4D) were performed at 45°C. It is seen that in the presence of calcium chloride, there is a gain in the tertiary interactions of holo-LA under crowded conditions, thus rendering holo-LA resistant from crowding-induced aggregation. Intrigued by the ability of calcium chloride in preventing holo-LA aggregation under crowded conditions, we further examined if calcium chloride could reverse preformed holo-LA aggregates by measuring the time dependent aggregation of holo-LA under crowded conditions using light scattering as the probe. It is seen in Figure 5 that upon addition of buffer to the saturated holo-LA aggregates, there was a slight decrease in the light scattering intensity at 400 nm, which is attributed to the dilution of holo-LA aggregates. While, upon addition of calcium chloride, the light scattering intensity reduced to zero, suggesting the efficacy of calcium in reversing the crowding-induced holo-LA aggregates. The ability of calcium to inhibit and reverse holo-LA aggregation led us to conclude that calcium might act as a chaperone *in vivo* at least for many calcium-binding proteins (CBPs). Similar to inducible chaperones, calcium (a signalling molecule) is also known to be upregulated under various proteopathic stress conditions (high temperature, low pH, oxidative stress) [55], [56], [57]. Indeed, the use of calcium as a chaperone has an added advantage over the molecular chaperones as the calcium chaperones do not require energy obtained from ATP hydrolysis to catalyze the dissociation of aggregates. Therefore, the use of calcium as a chaperone *in vivo* might have been evolutionarily more favored as compared to other molecular chaperones especially for CBPs.

As mentioned earlier, macromolecular crowding has been reported to have opposite consequences on the aggregation propensities of amyloidogenic (human Tau protein, human prion protein and human copper zinc superoxide dismutase) and non-amyloidogenic (rabbit prion protein and hen egg white lysozyme) proteins [32]. However, in this study, holo-LA which is a non-amyloidogenic protein is induced to aggregate by crowding agent. Furthermore, such a crowding-induced holo-LA aggregation is observed to be inhibited or reversed upon addition of CaCl_2_. In contrast to the protective role of calcium observed in this study, calcium has been reported to enhance the aggregation of A*β* (an amyloidogenic protein associated with AD) both *in vitro* and *in vivo* [58], [59], [60] and therefore, has been a risk factor for AD. The contrasting role of calcium in mediating amyloidogenic and non-amyloidogenic protein aggregation indicates that there might be existence of a group of CBPs in the neuronal cells to buffer increased intracellular calcium concentration under stressful conditions, so as to provide mutual benefit to A*β* and CBPs by offsetting aggregation of both. Interestingly, micro-array data of the neuronal cells from AD patients revealed that at least 6 genes involved in the expression of CBPs are down-regulated [61], [62] suggesting that the down-regulation of these CBPs might be one of the basic causes of the increased pathogenicity in AD (see Table 3). Thus we conclude that (i) increasing the expression of the calcium binding proteins might help to reduce AD pathogenesis by offsetting the A*β* aggregation; and (ii) calcium may mediate the aggregation of amyloidogenic and non-amyloidogenic proteins differently under crowded intracellular environment.

**Table 3:** List of genes downregulated in Alzheimer Disease.

Gene	Protein	References
CAMK2A	Calcium-dependent protein kinase II alpha	[61]
CAMK2G	Calcium-dependent protein kinase II gamma	[61]
PPP2CA	Protein phosphatase 2 catalytic subunit alpha isozyme	[61]
PPP3CA	Protein phosphatase 3 catalytic subunit alpha isozyme	[61]
PVALB	Parvalbumin	[61]
RGS4	Regulator of G protein signaling 4	[62]

## Supporting Information

Figure S1 **Temperature-dependent aggregation profiles of holo-LA.** The temperature dependence of the light scattering intensity at 400 nm of different concentrations of holo-LA in the presence of 400 g/l Ficoll 70. The lines represent the best-fits (using Equation 1) of data obtained in the presence of 50 µM (solid circle), 75 µM (solid triangle), 100 µM (solid inverted triangle), 150 µM (solid square) and 200 µM (solid diamond) holo-LA.
(TIF)

Figure S2 **Temperature-dependent aggregation profiles of apo-LA.** The temperature dependence of the light scattering intensity at 400 nm of apo-LA in the absence and presence of 400 g/l Ficoll 70. The lines represent the best-fits (using Equation 1) of data obtained in the presence of 0 g/l (solid circle) and 400 g/l Ficoll 70 (solid triangle).
(TIF)

Figure S3 **Light scattering measurements for Ficoll 70.** The light scattering intensity of 400 g/l Ficoll 70 alone as a function of time at 46.4°C.
(TIF)
