# Supplementary figures and images for: Macromolecular Crowding Induces Holo α-Lactalbumin Aggregation by Converting to Its Apo Form
Source: PLoS One. 2014 Dec 1;9(12):e114029. doi: 10.1371/journal.pone.0114029 (PMC4250181; doi:10.1371/journal.pone.0114029)

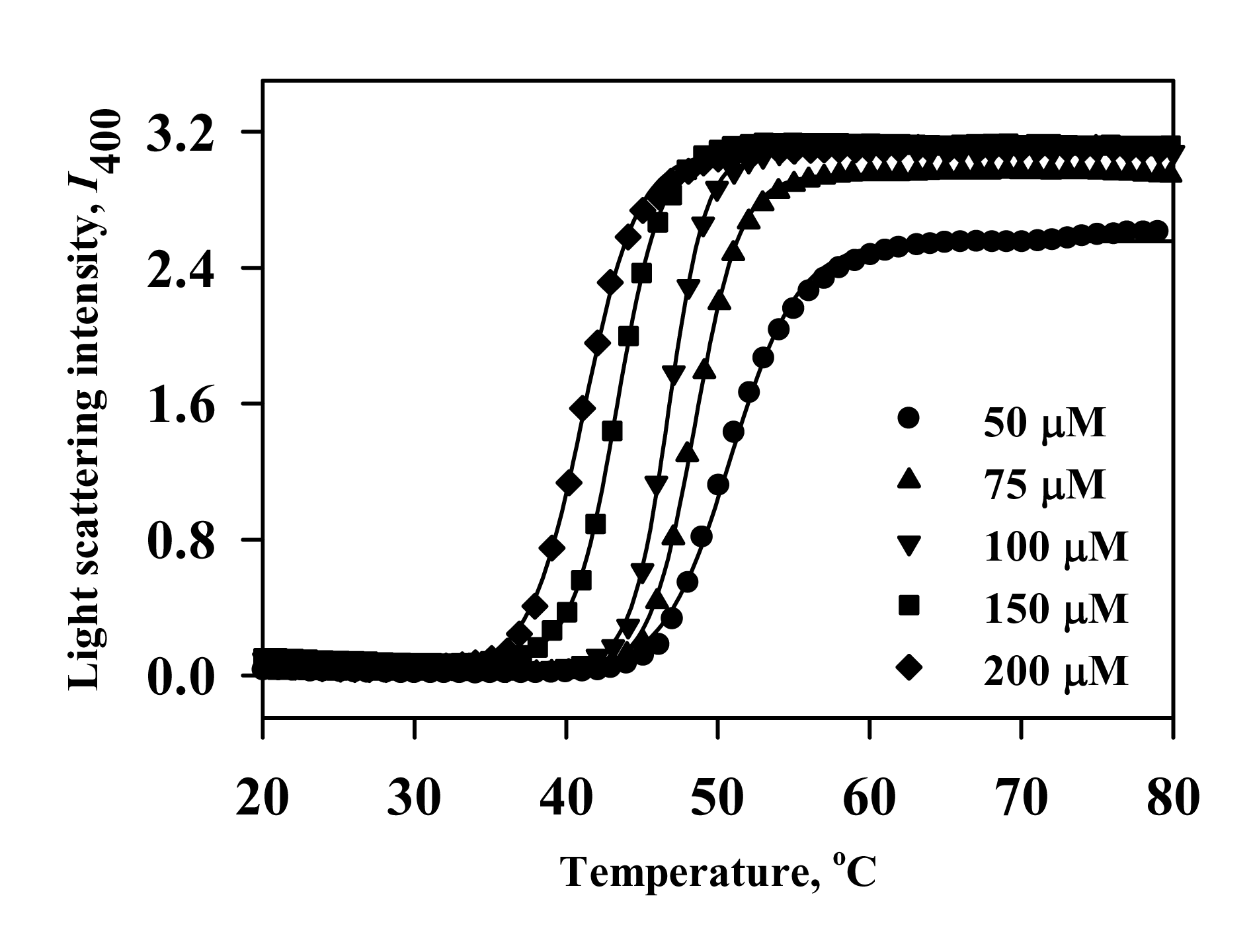

Supplement: Figure S1 — Temperature-dependent aggregation profiles of holo-LA. The temperature dependence of the light scattering intensity at 400 nm of different concentrations of holo-LA in the presence of 400 g/l Ficoll 70. The lines represent the best-fits (using Equation 1) of data obtained in the presence of 50 µM (solid circle), 75 µM (solid triangle), 100 µM (solid inverted triangle), 150 µM (solid square) and 200 µM (solid diamond) holo-LA. (TIF) [file pone.0114029.s001.tif]

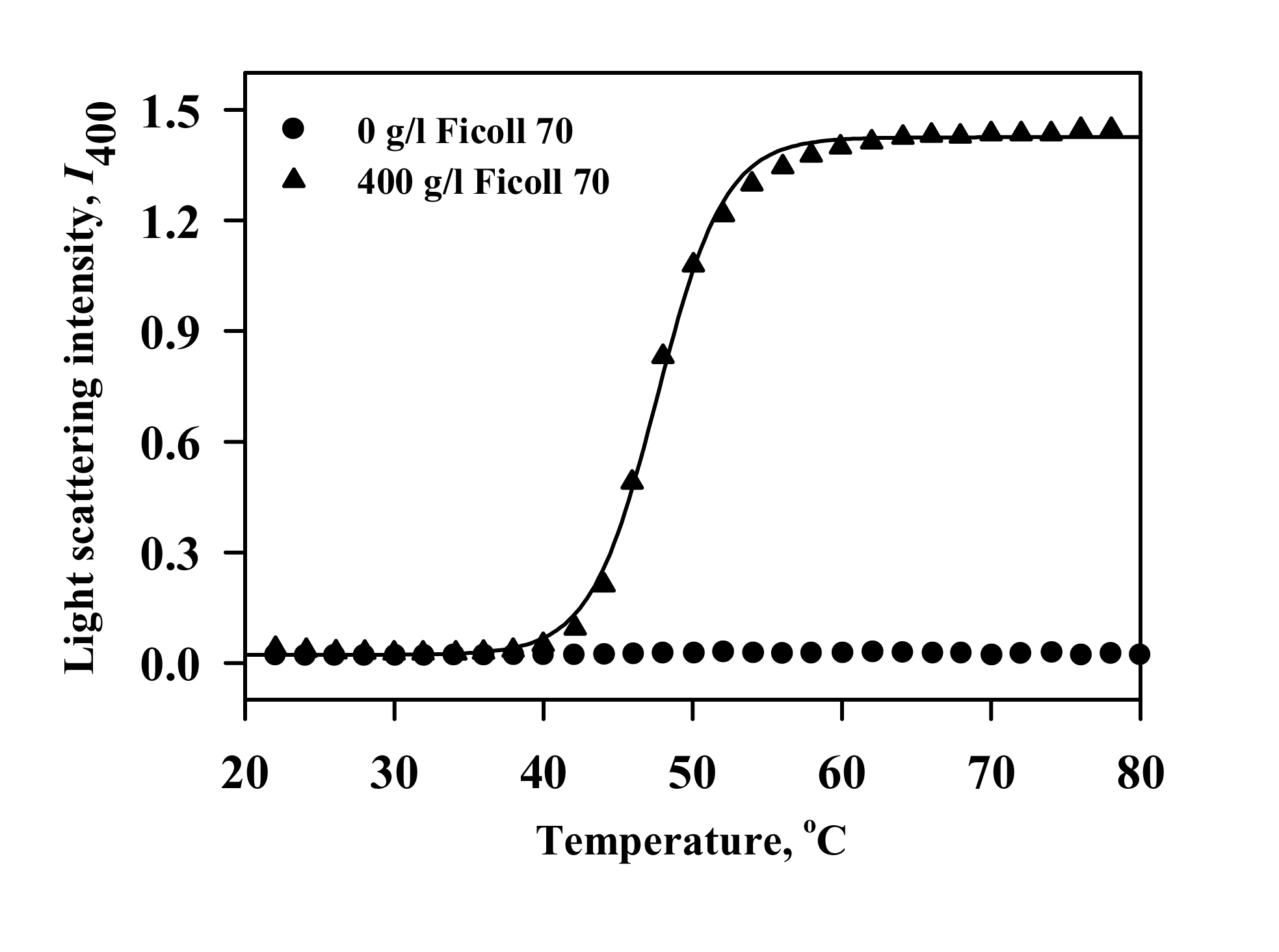

Supplement: Figure S2 — Temperature-dependent aggregation profiles of apo-LA. The temperature dependence of the light scattering intensity at 400 nm of apo-LA in the absence and presence of 400 g/l Ficoll 70. The lines represent the best-fits (using Equation 1) of data obtained in the presence of 0 g/l (solid circle) and 400 g/l Ficoll 70 (solid triangle). (TIF) [file pone.0114029.s002.tif]

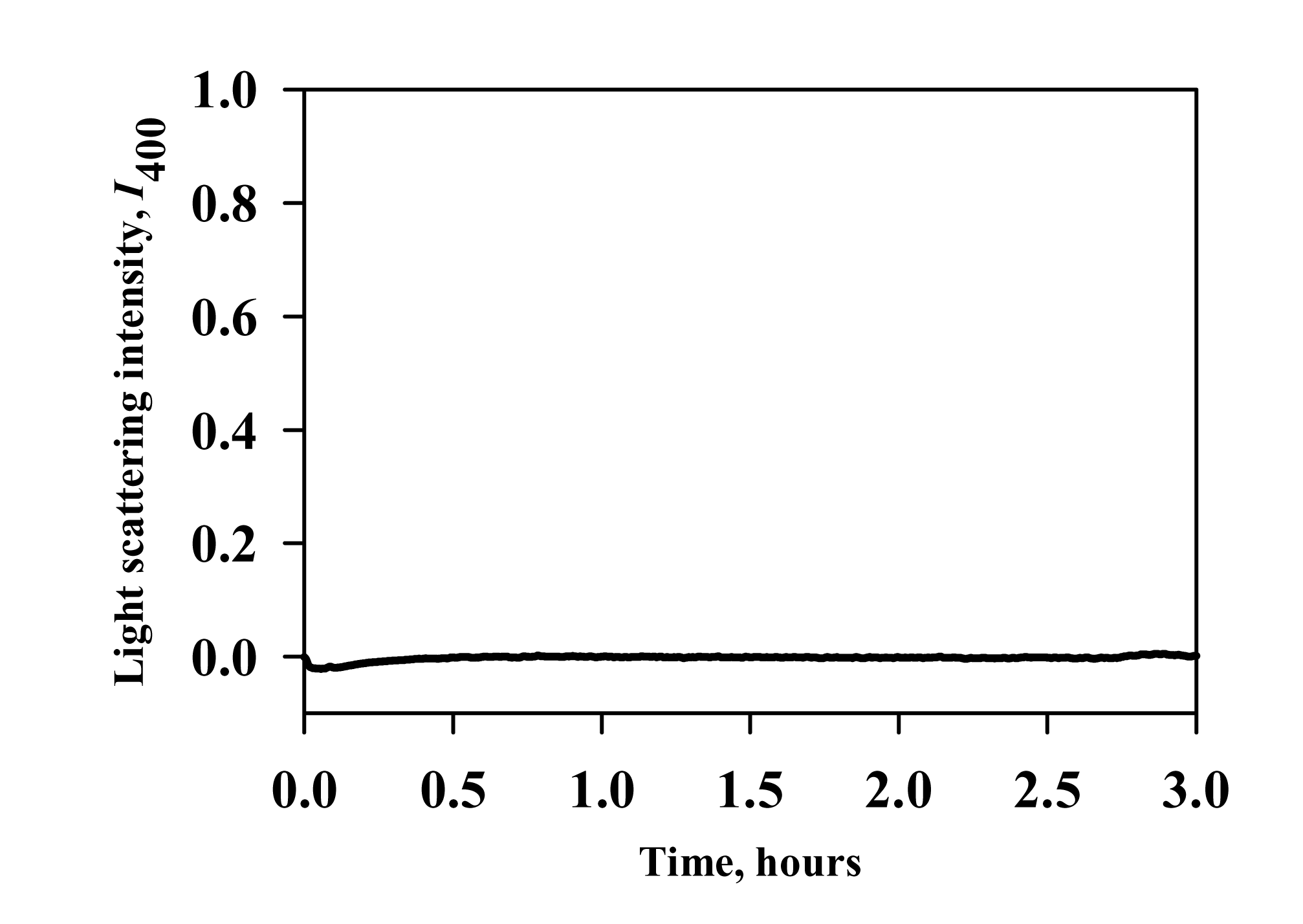

Supplement: Figure S3 — Light scattering measurements for Ficoll 70. The light scattering intensity of 400 g/l Ficoll 70 alone as a function of time at 46.4°C. (TIF) [file pone.0114029.s003.tif]
